# Multi‐omics studies reveal convergent and divergent pathways in Alzheimer's and related disorders

**DOI:** 10.1002/alz70855_096030

**Published:** 2025-12-23

**Authors:** Mariet Allen, Stephanie R Oatman, Xue Wang, Joseph S. Reddy, Thuy T. Nguyen, Dennis W. Dickson, Nilüfer Ertekin‐Taner

**Affiliations:** ^1^ Mayo Clinic, Jacksonville, FL, USA

## Abstract

**Background:**

Alzheimer's disease (AD) is pathologically characterized by the presence of extracellular amyloid beta plaques and intracellular tau tangles. Related disorders share aspects of AD pathology including progressive supranuclear palsy (PSP), a tauopathy, and pathologic aging (PA) defined by presence of amyloid but not tau. We hypothesize that there are shared, and distinct, brain molecular changes associated with amyloid and tau protein levels in AD and related disorders and that identifying these will provide novel insights into disease pathogenesis.

**Methods:**

We collected brain multiomics measures (genetic, transcriptomic, epigenomic, proteomic and metabolomic) from donors diagnosed with AD, PSP, PA and controls. Following quality control and normalization, omics measures were tested for association with disease diagnosis and amyloid and tau neuropathology using regression models adjusting for relevant covariates. Association with biochemical measures of core AD proteins were also assessed in a subset of AD cases. Gene sets were evaluated for enrichment of cellular pathways and cell type signatures.

**Results:**

We found remarkably conserved transcriptomic changes associated with AD and PSP, and downregulation of myelination pathway genes in both diseases. Among PSP cases, we found divergent patterns of transcriptional associations with distinct tau lesions. In a cohort of AD cases, we found broad transcriptional and epigenetic dysregulation associated with levels of tau protein, but not amyloid beta. Conversely a genetic analysis implicated more variants associated with amyloid levels than tau.

**Conclusion:**

Multi‐omics studies across various proteinopathies can identify convergent and divergent molecular signatures that are candidates for shared therapeutic targets and disease differentiating biomarkers.

